# Impact of human MLL/COMPASS and polycomb complexes on the DNA methylome

**DOI:** 10.18632/oncotarget.2215

**Published:** 2014-07-14

**Authors:** Emily L. Putiri, Rochelle L. Tiedemann, Chunsheng Liu, Jeong-Hyeon Choi, Keith D. Robertson

**Affiliations:** ^1^ Department of Molecular Pharmacology and Experimental Therapeutics and Center for Individualized Medicine, Mayo Clinic, Rochester, MN; ^2^ Cancer Center, Georgia Regents University, Augusta, GA

**Keywords:** DNA methylation, epigenetics, cancer, histone modification, histone methylation

## Abstract

The correlation between DNA methylation and a subset of histone post-translational modifications (positive and negative) has hinted at an underlying regulatory crosstalk between histone marks and DNA methylation in patterning the human DNA methylome, an idea further supported by corresponding alterations to both histone marks and DNA methylation during malignant transformation. This study investigated the framework by which histone marks influence DNA methylation at a genome-wide level. Using RNAi in a pluripotent human embryonic carcinoma cell line we depleted essential components of the MLL/COMPASS, polycomb repressive complex 2 (PRC2), and PRC1 histone modifying complexes that establish, respectively, the post-translational modifications H3K4me3, H3K27me3, and H2AK119ub, and assayed the impact of the subsequent depletion of these marks on the DNA methylome. Absence of H2AK119ub resulted predominantly in hypomethylation across the genome. Depletion of H3K4me3 and, surprisingly, H3K27me3 caused CpG island hypermethylation at a subset of loci. Intriguingly, many promoters were co-regulated by all three histone marks, becoming hypermethylated with loss of H3K4me3 or H3K27me3 and hypomethylated with depletion of H2AK119ub, and many of these co-regulated loci were among those commonly targeted for aberrant hypermethylation in cancer. Taken together, our results elucidate novel roles for polycomb and MLL/COMPASS in regulating DNA methylation and define targets of this regulation.

## INTRODUCTION

Cytosine methylation and histone post-translational modifications create a complex epigenetic system required for the regulation and maintenance of gene expression programs that specify cell state (i.e., pluripotent and differentiated cell states). Determining how CpG methylation and histone marks cooperate to instruct normal cell expression programs and how this cooperation breaks down in human disease is an important area only just beginning to be understood. Numerous histone modifications involved in transcriptional regulation have been identified [1]. Three such marks are monoubiquitination of histone H2A lysine 119 (H2AK119ub), histone H3K27 trimethylation (H3K27me3), and H3K4me3. H2AK119ub and H3K27me3 are typically linked to transcriptional repression and are mediated by the Polycomb group (PcG) Repressive Complexes, PRC1 and PRC2, respectively. PcG proteins regulate gene expression programs for developmental processes such as ESC pluripotency, cell fate decisions, and tissue patterning [2], and PcG binding and aberrant repression of tumor suppressor target genes facilitates cancer stem cell maintenance in many tumor types [3]. H3K27me3 is established by the histone methyltransferase enhancer of zeste homolog 2 (EZH2), but EZH2's catalytic activity is dependent on embryonic ectoderm development (EED) and suppressor of zeste 12 (SUZ12) [4, 5]. At some loci, PRC2 recruits PRC1 for ubiquitination of H2AK119, but PRC1 can function independently of PRC2 and H3K27me3 [6, 7]. PRC1 core components include RING1 & RING2 (RNF2), the RING-finger domain protein BMI1, and the polyhomeotic homologs PHC1-3 [8]; PRC1 ubiquitination of histone H2A is dependent on the E3-ubiquitin ligase subunit RING1B [6, 8]. In addition to PRC1's ubiquitination activity, PRC1 complexes may include one of several chromobox homologs, including CBX4, which possesses SUMO E3 ligase activity [9]. Although sumoylation targets of CBX4 are limited, known substrates include the transcriptional repressor C-terminal binding protein (CtBP) [9], DNA methyltransferase 3A (DNMT3A) [10], and BMI1 [11]. Sumoylation of CBX4 itself also facilitates binding of PRC1 to H3K27me3 [12]. H3K4me3 is associated with active transcription and is written by the MLL/COMPASS group of SET-domain histone methyltransferases, whose activities depend on interactions with WDR5, RBBP5, and ASH2 [13]. Gene promoters occupied by H3K4me3 generally lack repressive PcG marks, except at bivalent promoters in stem cells in which H3K4me3 and H3K27me3 coexist and are believed to facilitate transcriptional plasticity during differentiation [14].

Normal vertebrate cells display distinct patterns of genome-wide CpG methylation. Most CpGs exhibit a “default” methylated state, except in CpG islands (CGI), which are CpG-dense regions [15-17]. Many gene promoters are associated with CGI, which under normal conditions typically remain hypomethylated. Promoter CGI methylation is an impediment to transcriptional activity, and aberrant DNA hypermethylation is a key mechanism for tumor suppressor gene silencing in cancer cells. CpG methylation is established by the DNA methyltransferases, DNMT1, DNMT3A, and DNMT3B. DNMT3A and DNMT3B are *de novo* methyltransferases that establish new cytosine methylation patterns during cell fate specification [18]. DNMT1 maintains DNA methylation fidelity by copying CpG methylation patterns to the nascent strand during DNA replication [19].

Evidence suggests a functional crosstalk between DNA methylation and histone marks. Establishment of new DNA methylation patterns by DNMTs are histone modification-dependent: CpG-rich promoters marked by H3K27me3 in normal cells recruit DNMTs to induce *de novo* DNA methylation in cancer cells [20, 21], and H3K4me3 inhibits DNMT3L binding to the histone H3 N-terminus, an interaction required for DNMT3A and DNMT3B activity [22, 23]. Likewise, the presence of H3K4me3 is inversely correlated with DNA methylation at promoters of highly active genes [22]. Second, DNA methylation patterns provide feedback for histone modifing activities. EZH2 specifically binds and occupies unmethylated CGI [24], and globally hypomethylated DNMT1−/− cells display dramatic redistribution of H3K27me3 and loss of PRC2 occupancy at normal PcG target genes [25]. Finally, alterations in histone modification patterns in cancer cells correspond with aberrant DNA methylation patterns. For instance, loci with bivalent H3K4me3 and H3K27me3 marks in pluripotent stem cells are prone to acquiring promoter hypermethylation in progenitor cells that become transformed [14, 26]. Thus, histone modification occupancy and DNA methylation patterns are clearly interdependent; yet the mechanistic underpinnings of these relationships and whether they are direct or indirect remains unknown.

The goal of this study was to determine how histone modifications functionally interface with, or regulate, genome-wide CpG methylation patterns. We interrogated the functional impact of MLL/COMPASS, PRC1, and PRC2 complexes on the human DNA methylome as these three marks have the strongest associations with DNA methylation. Our results show that both MLL/COMPASS and PRC2 prevent DNA methylation, predominantly at CGI and promoters. PRC1 promotes DNA methylation at CGI and many of the same promoter targets regulated by MLL/COMPASS and PRC2. Overall, MLL/COMPASS and polycomb complexes epigenetically regulate loci susceptible to DNA hypermethylation in cancer cells, and the opposing activities of PRC1 and MLL/COMPASS and PRC2 likely serve to establish and/or maintain a chromatin environment that finely regulates both pro- and anti-DNA methylation recruitment signals at these epigenetically metastable loci. This study constitutes a comprehensive genome-wide analysis of the impact of MLL/COMPASS and PcG functions on DNA methylation and unveils an unexpected role for PRC2 in preventing CGI hypermethylation that may have important mechanistic implications for how abnormal DNA methylation patterns arise in tumors, especially those driven by mutations in epigenetic modifiers.

## RESULTS

### MLL/COMPASS activity protects CpG islands and bivalent promoters from DNA hypermethylation

**Figure 1:**
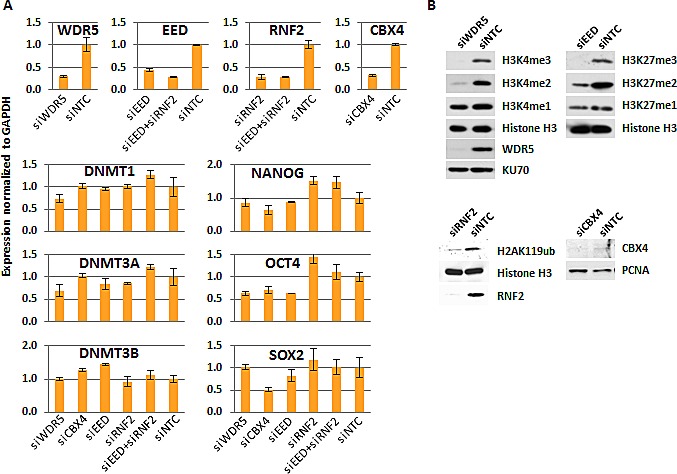
Validation of the approach for H3K4me3, H3K27me3, H2AK119ub, and CBX4 depletions (A) Gene expression measured by QRT-PCR normalized to GAPDH and relative to siNTC for each targeted siRNA depletion. Expression of the DNMTs and pluripotency regulators in each siKD condition is also shown. (B) Western blot of cell extracts from targeted-siRNA depleted cells and siNTC control treated cells for histone marks and protein targets as indicated. EED western blot is not shown because the antibody failed to detect a single, specific band (not shown).

To study the impact of MLL/COMPASS on DNA methylation, we targeted WDR5, a component essential for all MLL/COMPASS complexes to bind the histone H3 N-terminal tail [27], for siRNA-mediated depletion using the human embryonic carcinoma cell line (ECC) NCCIT as a model system. NCCIT is a nonseminomatous germ cell-derived teratoma that exhibits characteristics of pluripotent cells, in that it can be induced to differentiate into embryonic germ layers, and the NCCIT transcriptome resembles that of human embryonic stem cells (ESC) [28, 29]. Depletion of H3K4 methylation has previously been shown to increase total genomic cytosine methylation levels using *Saccharomyces cerevisiae* as a model system [22], but the existence of a direct link between H3K4me and DNA methylation in the human genome remains unknown. We therefore asked whether depletion of H3K4me3 by siRNA against WDR5 (siWDR5) would result in DNA hypermethylation, if indeed these two epigenetic marks are functionally interrelated. A non-targeting control siRNA (siNTC) was used for comparison. Transfection with siWDR5 generated ~70% depletion of the target mRNA (Fig. 1A) and >95% depletion of the target protein (Fig. 1B). Depletion of WDR5 eliminated H3K4me3, and reduced H3K4me2 dramatically (Fig. 1B). Total H3K4me1 was unaffected by WDR5 depletion (Fig. 1B). Treatment with siWDR5 had little impact on expression of DNMTs or pluripotency factors (Fig. 1A).

**Figure 2:**
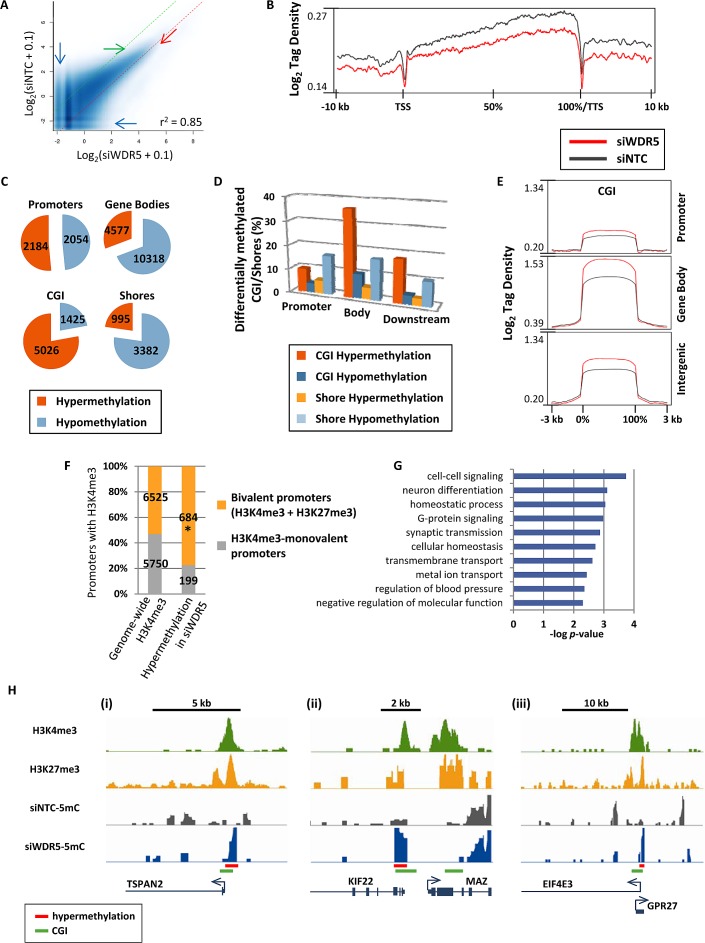
Disruption of MLL/COMPASS complex and H3K4 methylation induces DNA hypermethylation at CGI and bivalent promoters (A) Scatterplots for peaks of 5mC called by SICER [41] in siWDR5 treated cells. Methylation enrichment levels for all sequenced loci are plotted on the X-axis (for siWDR5 treatment) and Y-axis (for siNTC). Dotted green and red lines represent limits for 2-fold hypomethylation and hypermethylation, respectively. Pearson's correlation coefficient is shown. (B) 5mC-tag density plot across all genes from −10 kb upstream of the transcription start site (TSS) to +10 kb downstream of the transcription termination site (TTS). Genes are normalized for length and shown as a percentage with 0% as the TSS and 100% as the TTS. (C) Pie charts for genes with decreased and increased 5mC in promoters, gene bodies, CGI, and CGI shores. Pie pieces represent the total number of genetic features with 2-fold or greater hypermethylation or hypomethylation. (D) Bar graph showing the proportion of CGI and shores in promoters, gene bodies, and gene 3'ends that become hyper- or hypomethylated in siWDR5 treated cells. (E) Log_2_ tag density plots of methylation for CGI in promoters, gene bodies, and intergenic regions. (F) Graph illustrates the proportion and number of H3K4me3-monovalent and bivalent promoters within the entire genome and those that become hypermethylated specifically in siWDR5-treated cells. * indicates that the proportion is greater than expected with p < 0.0001. (G) Ontology analysis of siWDR5-hypermethylated bivalent genes. (H) Examples of bivalent promoters that become hypermethylated in siWDR5-treated cells: *TSPAN2* (i), *MAZ* (ii), and *EIF4E3* (iii). Top two tracks are from previously published ChIP-seq data in unperturbed NCCIT [17]; bottom tracks are 5mC data from siRNA knockdown samples. All 5mC-seq tracks are presented on the same y-axis scale (minimum and maximum y-axis values are: 0 to 1.11 for *TSPAN2*, 0 to 1.21 for *MAZ*, and 0 to 1.21 for *EIF4E3*).

Affinity purification of methylated DNA using the methyl binding domain of MBD2, followed by high-throughput sequencing (5mC-seq) was used to identify fragmented genomic DNA enriched in CpG methylation [17], and peaks of DNA methylation were called for siWDR5 and siNTC treated cells. Scatterplots of methylation levels for all identified peaks revealed both hypo- and hypermethylation of many loci with low basal levels of methylation (Fig. 2A, blue arrows), but loci with moderate to high levels of methylation tended to become hypermethylated (in Fig. 2A note the “clearing” marked by the green arrow, and the increased methylation marked by the red arrow). 5mC tag density plots across gene bodies revealed an overall hypomethylation effect on the genome resulting from WDR5 depletion (Fig. 2B). Thus, hypermethylation observed in the scatterplots must affect either a subset of genes/features or intergenic regions. Indeed, after counting genes with differentially methylated peaks, more than 10,000 genes showed hypomethylation in gene bodies, but a large proportion of gene promoters were hypermethylated (Fig. 2C). CGI showed robust hypermethylation (Fig. 2C). Nearly 10% of promoter CGI were hypermethylated and 36% of intragenic CGI were hypermethylated (Fig. 2D,E). In contrast to CGI, CGI shores became mostly hypomethylated upon siWDR5-depletion, and CGI shores in promoters or gene bodies were equally impacted (Fig. 2D). Since inhibition of WDR5 impacts H3K4me3, we examined the relationship between hypermethylated promoters in siWDR5-treated cells and promoters marked with H3K4me3 determined by previously published NCCIT H3K4me3 ChIP-seq data [17]. Only a subset of siWDR5 hypermethylated promoters had H3K4me3 (883 out of 2184) prior to WDR5 knockdown. Of these hypermethylation events, 77% (24% greater than expected) occurred at bivalent H3K4me3+H3K27me3 loci, but H3K4me3 monovalent promoters were underrepresented among hypermethylated H3K4me3-marked promoters in siWDR5 cells (Fig. 2F). Bivalent promoters impacted by DNA hypermethylation in WDR5-depleted cells included genes with functions involved in cell-cell signaling, G-protein signaling, neuronal fate, and synaptic transmission (Fig. 2G), and included genes such as those encoding the transmembrane protein *TSPAN2*, the oncogene *MAZ*, and the tumor suppressor gene *EIF4E3* (Fig. 2H). We sought to further independently confirm our novel findings related to siWDR5-induced DNA hypermethylation, therefore we applied siNTC and siWDR5 DNA to the Infinium 450k array, which interrogates methylation at single CpG resolution using bisulfite conversion at sites spread throughout the genome [30]. Results from this experiment demonstrate that CGI hypermethylation in siWDR5 detected by the Infinium 450k array is a significant subset of that detected by 5mC-seq. Furthermore, the 5mC-seq observation for siWDR5 in which hypomethylation is predominantly observed at non-CGI loci and hypermethylation is observed at CGI is supported by the Infinium 450k results (Supp. Fig. 1). Methylated DNA immunoprecipitation (MeDIP) coupled to qPCR was also used as validation of the 5mC-seq results, and further confirms several WDR5 depletion-induced DNA methylation changes predicted by the 5mC-seq (Supp. Fig 2). Loci that lost DNA methylation upon WDR5 depletion tended not to be enriched for H3K4me3, but rather were more linked to the presence of H3K27me3 and H2AK119ub (Supp. Fig 3A). In summary, the presence of H3K4 methylation inhibits DNA methylation specifically at promoters and CGI, and this inhibitory function is strongest at bivalent promoters compared to monovalent H3K4me3 promoters. Hypomethylation upon WDR5 knockdown may result from redistribution of the DNA methylation machinery from repressed PcG-marked loci to regions previously protected by H3K4me3. These data therefore demonstrate an active role for H3K4me3 in restricting DNA methylation within certain regions of the genome.

### PcG complexes promote and inhibit DNA methylation; PRC2 specifically protects CGI from DNA hypermethylation

**Figure 3:**
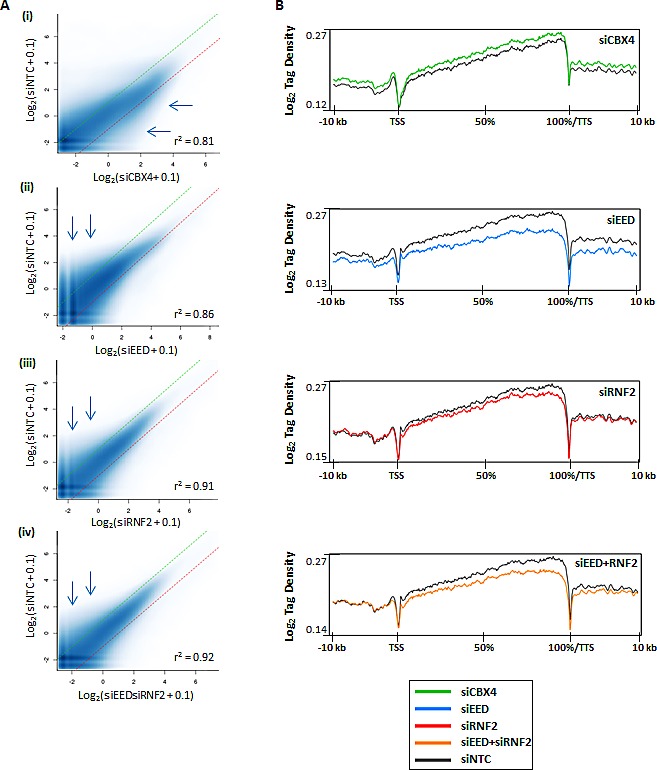
Differential DNA methylation in polycomb complex function-depleted cells (A) Scatterplots for peaks of 5mC called by SICER [41]. Methylation enrichment levels for all sequenced loci are plotted on the X-axis (for siRNA treatment) and Y-axis (for siNTC). Dotted green and red lines represent limits for 2-fold hypomethylation and hypermethylation, respectively. Pearson's correlation coefficient is shown. (B) 5mC-seq tag density plots across all genes from -10 kb upstream of the transcription start site (TSS) to +10 kb downstream of the transcription termination site (TTS). Genes are normalized for length and shown as a percentage with 0% as the TSS and 100% as the TTS.

**Figure 4:**
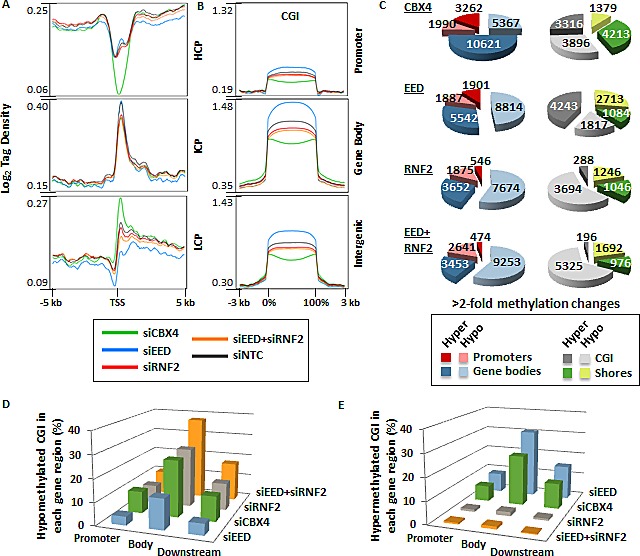
Impact of PRC1 and PRC2 depletion on DNA methylation patterns (A) Tag density plots of methylation from −5 to +5 kb across gene promoters at high CpG density loci (HCP), intermediate CpG density loci (ICP), and low CpG density loci (LCP) as defined in [43]. (B) Tag density plots of methylation across CGI located in promoters, gene bodies, and intergenic regions. (C) Pie charts for genes with >2-fold decreased and increased 5mC in promoters, gene bodies, CGI, and CGI shores. (D-E) Bar graphs illustrate the proportion of CGI in promoters, gene bodies, and downstream of genes that become hypomethylated (D) or hypermethylated (E) in each siRNA depletion.

To understand the impact of PcG function on DNA methylation globally, we again used siRNA to deplete a component essential to each PcG complex's catalytic activity. SiRNA against EED was used to inhibit PRC2 activity, RNF2 siRNA was used to target the ubiquitination activity of PRC1, and CBX4 was targeted by siRNA to elucidate the impact of its sumoylation activity (or other possible functions like DNMT interaction [10, 31]) on DNA methylation. Each siRNA treatment yielded 55-75% reduction of the targeted mRNA (Fig. 1A). SiRNA treatments were assessed for their impact on changes in DNMT expression, which remained relatively stable (Fig. 1A). Pluripotency markers were also unaffected, except for SOX2 expression, which was reduced by half in siCBX4 treated cells (Fig. 1A). Treatment of NCCIT cells with siEED nearly eliminated H3K27me3, H3K27me2 was partially depleted, and H3K27me1 was unaffected relative to the siNTC control (Fig. 1B). SiRNF2 treatment reduced H2AK119ub by more than 80% and siCBX4 successfully reduced both its mRNA and protein level (Fig. 1). Following 5mC-seq analysis of each of these siKDs, we observed that CBX4 depletion caused an overall trend toward hypermethylation throughout the genome (Fig. 3A,i, blue arrows), which was readily apparent across gene bodies (Fig. 3B). The exception to this overall siCBX4-induced DNA hypermethylation trend occurred at high CpG-density promoters (HCP), CGI, and CGI shores, which lost DNA methylation (Fig. 4A-C). Treatment with siEED resulted predominantly in hypomethylation of loci with low to moderate basal levels of methylation (Fig 3A,ii, blue arrows) but caused hypermethylation at a subset of loci. On average, gene bodies and promoters with low CpG density were hypomethylated under these conditions, but CGI and a subset of high CpG density promoters were targeted for DNA hypermethylation in siEED-cells (Fig. 4A-C). Reduction of RNF2 or a combination of EED and RNF2 resulted in widespread hypomethylation throughout the genome, including gene bodies and CGI (Fig. 3A,B; Fig. 4A-C). The result that dual siEED+siRNF2 depletion was similar to siRNF2 single depletion suggests that RNF2 depletion suppresses the hypermethylation effect of EED depletion. Across all siRNA depletion samples (CBX4, EED, RNF2, and EED+RNF2), CGI within gene bodies experienced the highest proportion of hypo- and hypermethylation events compared with promoter CGI and CGI located to the 3'end of genes (Fig. 4D,E). In summary, CBX4 functions to limit DNA methylation levels while EED and RNF2 generally promote DNA methylation, except at CGI where CBX4 promotes DNA methylation and EED (and by inference H3K27me3) promotes a hypomethylated state.

### PcG complexes have the greatest impact on DNA methylation at loci marked by both H3K27me3 and H2AK119ub

**Figure 5:**
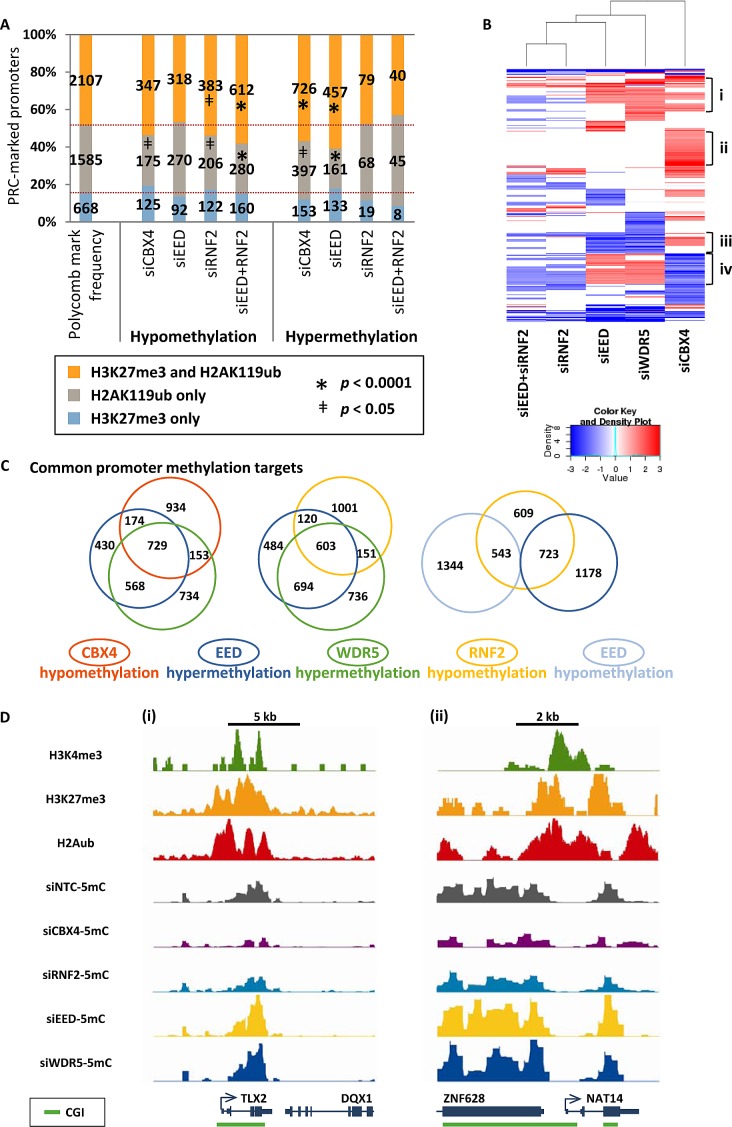
Selective epigenetic co-regulation of DNA methylation at target genes by PcG complexes (A) Graph illustrates the frequency and number of promoters marked exclusively with H3K27me3, marked exclusively with H2AK119ub, or marked with both H3K27me3 and H2AK119ub. Shown are the frequencies of these promoters among all promoters in the genome and among those promoters that become hypo- or hypermethylated in each of the siRNA knockdown samples in NCCIT cells. Promoters with methylation changes that are over- or underrepresented among those with the given chromatin marks are designated with * when *p* < 0.0001 or ǂ when *p* < 0.05. (B) Heat map of hierarchical clustering of promoter hypomethylation and hypermethylation events shown as log_2_ methylation changes. (C) Area proportional Venn diagrams illustrating overlapping promoter hypermethylation and hypomethylation events. *p* < 0.0001 for all depicted overlapping subsets using a two-tailed Fisher's exact test. (D) Examples of promoters in the (i) *TLX2* and (ii) *NAT14* genes that are hypomethylated in siRNF2 and siCBX4 samples but hypermethylated in siEED and siWDR5 samples. Top three tracks are from previously published ChIP-seq data in unperturbed NCCIT cells [17]; bottom tracks are 5mC-seq data from siRNA KD samples. All 5mC-seq tracks are presented on the same y-axis scale (minimum and maximum y-axis values are: 0 to 7.43 for *TLX2*, and 0 to 6.60 for *NAT14*).

We next investigated the loci targeted by PcG complexes in greater detail. Given that disruption of PcG complexes impacts PRC targeted histone marks (Fig. 1B), we asked whether hyper- and hypomethylation events occurred at loci with these marks. Hypomethylation in siRNF2 and in siEED+RNF2 treated cells was more likely to occur at loci with both PRC1 and PRC2 marks (H3K27me3 and H2AK119ub) and less likely to occur at loci marked with only H2AK119ub (Fig. 5A). Similarly, hypermethylation induced by siCBX4 or siEED treatments disproportionally impacted loci with both H3K27me3 and H2AK119ub (Fig. 5A). H3K4me3 monovalent promoters were anti-correlated with promoter hypo- or hypermethylation, but bivalent promoters were not significantly susceptible to nor excluded from PRC-depletion-induced methylation changes (Supp. Fig. 3B). Thus, H3K27me3 and H2AK119ub together create a chromatin landscape predisposed, or permissive, to DNA methylation changes, where depletion of RNF2 is sufficient for DNA hypomethylation and depletion of EED function permits DNA hypermethylation (albeit only in the presence of functional RNF2).

The finding that loci with both H3K27me3 and H2AK119ub were most susceptible to DNA methylation changes in all PcG knockdowns suggested that common targets among the samples might exist. Hierarchical clustering revealed several subsets of promoters targeted for DNA hypo- and hypermethylation (Fig. 5B). Four clusters stand out among the differentially methylated promoters. First is a cluster of promoters that are hypermethylated in two or more of siEED, siCBX4, or siWDR5 depletions (Fig. 5B,i); these genes are enriched for processes involved in cell-cell signaling and glycosylation (Supp. Fig. 3C,i). Second is a large set of promoters exclusively hypermethylated by siCBX4 treatment (Fig. 5B,ii), which are involved in neuromuscular biology and post-embryonic development (Supp. Fig. 3C,ii). A third set of promoters become hypomethylated in both siEED and siWDR5 depletions (Fig. 5B,iii), and these are characterized by genes involved in chromosome condensation (Supp. Fig. 3C,iii). Last is an intriguing subset of promoters hypomethylated in siCBX4 and/or siRNF2 treatments but hypermethylated in siEED and/or siWDR5 treatments (Fig. 5B,iv). This last set of genes is representative of processes such as germ cell and reproductive development and meiosis (Supp. Fig. 3C, iv). In general, promoter hypomethylation induced by either siCBX4 or siRNF2 treatment was strongly associated with hypermethylation induced by siEED or siWDR5, suggesting co-regulation of DNA methylation by PcG and MLL/COMPASS complexes at a common set of target promoters (Fig. 5C). Furthermore, a significant proportion of hypomethylated promoters in siRNF2-treated cells were either hypo- or hypermethylated under EED siRNA depleted conditions suggesting functional co-targeting by PRC1 and PRC2 (Fig. 5C). Co-regulated genes included N-acetyltransferase 14 (*NAT14*) and T-cell leukemia homeobox 2 (*TLX2*), which were hypermethylated by loss of WDR5 or EED, but hypomethylated under conditions of CBX4 or RNF2 depletion (Fig. 5D). Thus overall, polycomb repressive and MLL/COMPASS complexes co-regulate the epigenetic landscape of a common set of target genes, where EED and WDR5 prevent DNA hypermethylation and RNF2 and CBX4 promote DNA methylation.

### Select polycomb complex-repressed genes experience promoter hypomethylation and gene activation upon PcG depletion

Microarray expression analysis was performed for all siRNA depletion conditions to assess the impact of MLL/COMPASS and PRC1/PRC2 perturbations on gene expression (Supp. Fig. 4A). All siRNA knockdown conditions resulted in both gene activation and repression events. EED or RNF2 depletion resulted in more gene activation than repression, consistent with their established PcG repressive functions, while dual EED + RNF2 depletion yielded similar numbers of activated and repressed genes (Supp. Fig. 4A). Interestingly, a large proportion of targets in the individual siEED or siRNF2 knockdowns overlapped, but a large, unique set of target genes were silenced upon dual EED + RNF2 knockdown (Supp. Fig. 4B). CBX4 and WDR5 depletion resulted in more transcriptional repression than activation events (Supp. Fig. 4A). To determine whether transcriptional changes were connected to DNA methylation changes, we compared the transcriptionally regulated and epigenetically regulated targets within each siRNA knockdown. Overall, expression changes did not correlate significantly (positively or negatively) with DNA hypo- or hypermethylation events (data not shown), but of genes that became activated under siEED or siRNF2 depletion conditions, a large proportion experienced promoter hypomethylation (Supp. Fig. 4C). Activated genes with hypomethylated promoters under siEED or siRNF2 depletion conditions were enriched for functions involved in epidermis development, kinase activation, and cell growth (Supp. Fig. 4D). Thus, many developmental genes that are targeted by PcG and MLL/COMPASS are regulated independently of DNA methylation, but a subset of target genes are epigenetically co-regulated via DNA methylation, which we suspect provides more rigid and stable epigenetic control in the context of PcG and MLL/COMPASS regulation.

### PRC2 and MLL/COMPASS complexes inhibit DNA methylation at genes commonly targeted for hypermethylation in cancer

**Figure 6:**
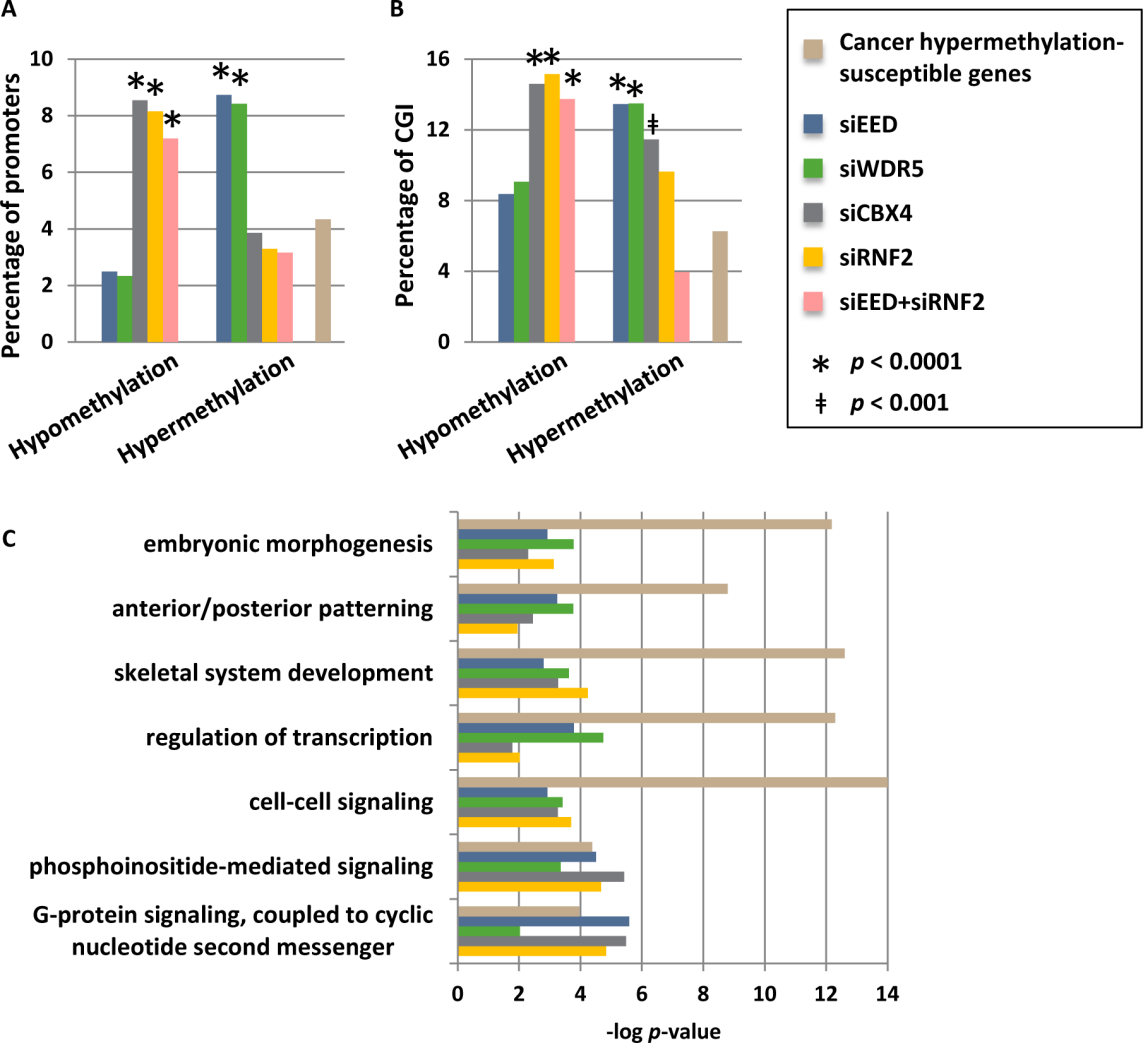
Promoters that are hypermethylated by depletion of WDR5 or EED are commonly hypermethylation in cancer (A) Genes with hypomethylated and hypermethylated promoters, or (B) CGI-containing promoters only after siRNA depletions were compared to genes previously identified as being commonly susceptible to hypermethylation in human cancers (n=1009; [32]). Shown are the percentages of differentially methylated genes for each sample that are also in the list of genes susceptible to cancer hypermethylation, and the percentage of total genes within the genome that are susceptible to cancer hypermethylation (total number of genes = 23218). Promoters with methylation changes that are overrepresented or underrepresented among the cancer hypermethylation list are designated with * when *p* < 0.0001 or ǂ when *p* < 0.001. (C) Ontology analysis of subsets of cancer hypermethylated genes that become differentially methylated under our siRNA knockdown conditions.

Given that bivalent H3K4me3 and H3K27me3-marked promoters are prone to acquiring aberrant DNA hypermethylation during cellular transformation [14, 26], coupled with our finding that PRC2 and MLL/COMPASS function to restrict DNA methylation, particularly at CGI, we asked whether loci commonly hypermethylated in cancer cells are disproportionately hypermethylated in our MLL/COMPASS- and PRC1/PRC2-depleted cells. A set of 1009 genes have been identified as commonly hypermethylated across a wide spectrum of human cancers [32]. These cancer hypermethylated genes were compared to those that sustain promoter DNA hypo- and hypermethylation changes in our siRNA knockdown conditions. Promoters that become hypermethylated by depletion of EED or WDR5 were more than twice as likely to be represented among genes hypermethylated in cancer (Fig. 6A). Similarly, genes that become hypomethylated by functional depletion of CBX4 or RNF2, or by joint knockdown of EED and RNF2, are enriched for those that commonly become hypermethylated in human cancers (Fig. 6A). These connections were present when comparing all promoters or just promoters containing CGI (Fig. 6B). Breakdown of the commonly hypermethylated in cancer gene set by the tumor types from which it was generated (breast, colorectal, prostate, lung, brain (glioblastoma), ovarian, and acute myelogenous leukemia (AML)) revealed similar associations with breast, prostate, lung, and AML tumor types (albeit with reduced significance due to smaller numbers of genes in each list) but not colorectal, glioblastoma, or ovarian tumors (data not shown). Genes susceptible to cancer hypermethylation that sustained epigenetic changes in our siRNA depleted cells were particularly enriched in embryonic patterning and morphogenesis, cell signaling pathways, and transcription; pathways commonly targeted for aberrant epigenetic silencing in human cancers [26] (Fig. 6C). In summary, MLL/COMPASS and PRC2 promote a hypomethylated state, particularly focused on promoters and CGI, while PRC1 promotes DNA hypermethylation; promoters where this interplay occurs are susceptible to aberrant DNA hypermethylation in human cancers.

## DISCUSSION

Several lines of evidence suggest the existence of functional crosstalk between DNA methylation and chromatin modifications [14, 20-22, 24-26, 33], and disruption of these interactions is intimately linked to cancer initiation and progression. This study comprised the first systemic investigation into the genome-wide function of MLL/COMPASS, PRC2, and PRC1 complexes in limiting or promoting DNA methylation in human cells. MLL/COMPASS and PRC2 predominantly opposed DNA methylation at CGI and promoters. RNF2's E3 ubiquitin ligase activity in PRC1 promotes methylation, but PRC1's CBX4 subunit has dual roles in promoting methylation (at CGI) and restricting methylation (across most gene regions). Importantly, loci most impacted by both DNA hypermethylation (in EED or WDR5 depletions) and hypomethylation (in CBX4 and RNF2 depletions) overlapped extensively among the knockdowns indicating multi-layered epigenetic co-regulation, and these loci were strongly susceptible to aberrant hypermethylation in cancer. Whether MLL/COMPASS and the PcG complexes have a similar impact on the methylome of other cell types as they do in NCCIT EC cells represents an interesting topic for future studies, however our finding that many of the same genes affected in the NCCIT cell model system are also affected in primary human tumors, suggests that our results have broader applicability.

Since DNA methylation is correlated with the absence of H3K4 methylation, and since methylation of the K4 residue impairs DNMT3L interaction with chromatin (an interaction that facilitates DNMT3A and DNMT3B enzymatic activity) [22], we anticipated that H3K4me3 would prevent DNA methylation throughout the genome. Our results show that, indeed, H3K4me3 prevents hypermethylation at CGI and bivalent promoters; however perturbation of MLL/COMPASS did not result in considerable hypermethylation at other loci. This may be due to the default methylated state of CpGs outside of CGI or the presence of the CpG-binding CXXC domain within the MLL1 protein and within the H3K4 methyltransferase SET1 interacting partner CFP1, which targets the MLL/COMPASS complex primarily to unmethylated CGI [34, 35]. Hypomethylation events under MLL/COMPASS-depletion conditions may stem from global redistribution of DNMTs upon loss of H3K4me3 protected zones. Indeed, precedence for global redistribution of one epigenetic mark upon depletion of another exists [25].

The gene repressive functions of PRC1 and PRC2 during embryonic development have been known for decades [36], but their enzymatic activities have only just begun to be elucidated, and the mechanisms by which they mediate repression remain vague. PRC2 has long been thought to mediate repression through recruitment of DNMTs [20, 21], but this function may apply to a subset of loci, since only a small proportion of activated genes were hypomethylated under PRC2 depletion conditions. More recently, the CXXC-domain-containing catalytic subunit of PRC2, EZH2, was shown to bind preferentially to hypomethylated CGI [24], and peaks of H3K27me3 occupancy have been associated with transcriptional activity [37]. Our novel finding that functional perturbation of PRC2 leads to DNA hypermethylation (in addition to hypomethylation events) at a subset of genes and that this hypermethylation is significantly associated with loci that become hypermethylated in the cancer state, sheds important light on the multi-faceted roles of PRC2. As evidenced by our data, PRC2 impacts DNA methylation in both directions—hypo- and hypermethylation—but the hypermethylation effect, which predominated at CGI and promoters, was unexpected because previous studies of dominant-negative H3.3 K27M mutant glioblastomas, which have an extensive global loss of H3K27me3, reported only a DNA hypomethylation impact on the genome [33].

**Figure 7:**
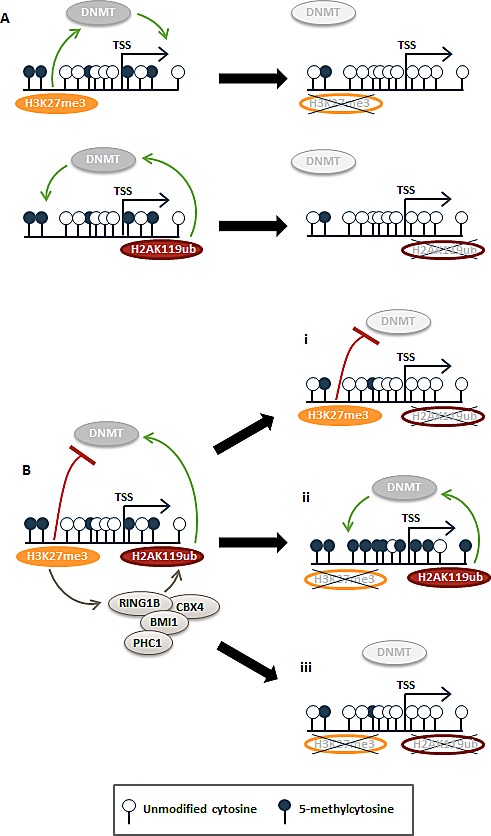
Model for how H3K27me3 and H2AK119ub epigenetic signatures modulate DNA methylation patterns (A) Two distinct subsets of genes were hypomethylated in the single siEED or siRNF2 knockdown conditions. We propose that these are loci in which PRC1 and PRC2 function independently of one another to influence DNA methylation. In the absence of PRC1 or PRC2, these loci become hypomethylated. (B) At another subset of genes, PRC2 and PRC1 act in concert to create a metastable epigenetic state. At these loci, H3K27me3 recruits PRC1 to deposit H2AK119ub. Together these marks establish opposing influences on DNA methylation: H2AK119ub promotes DNA methylation, presumably via DNMT recruitment, but H3K27me3 inhibits DNMT recruitment. With depletion of H2AK119ub, low levels of DNA methylation/DNMT recruitment are lost (i). If H3K27me3 is depleted at previously H3K27me3-marked loci, H2AK119ub remains unrestrained and promotes enrichment of DNA methylation (ii). In the event that both H3K27me3 and H2AK119ub are depleted, recruitment of DNMTs is reduced or lost.

We show that PRC2's role (and by inference H3K27me3's role) in repressing DNA methylation preferentially occurs at loci with both H3K27me3 and H2AK119ub marks, indicating that chromatin configuration impacts PRC2 regulation of DNA methylation. We propose a model wherein H3K27me3 and H2AK119ub may function either separately (Fig. 7A) or together (Fig. 7B) in regulating DNA methylation. Two independent sets of loci were found to be hypomethylated with either H3K27me3 depletion or with H2AK119ub depletion (Fig. 5C), and we suspect PcG marks are functioning independently of one another to promote CpG methylation at these loci. Depletion of the predominant mark is sufficient for loss of CpG methylation (Fig. 7A). On the other hand, at loci where hypermethylation occurs in the absence of H3K27me3 but hypomethylation occurs with depletion of H2AK119ub, we propose a separate scenario that is supported by the canonical model for PRC1/H2AK119ub recruitment via localization of PRC2/H3K27me3 [6, 7] (Fig. 7B). Deposition of both DNA methylation-preventing H3K27me3 and DNA methylation-promoting H2AK119ub at a given locus generates a balanced, but metastable epigenetic signature—a state of transcriptional plasticity that is more easily modulated during development or in response to fluctuating environmental stimuli (Fig. 7B). If H2AK119ub is removed at these loci, H3K27me3 protects the region from CpG methylation resulting in a hypomethylated state (Fig. 7B,i). In the event of H3K27me3 loss (during normal development or aberrantly during malignant transformation), PRC1 (and by inference, H2AK119ub) promotes deposition of DNA methylation (Fig. 7B,ii). Our finding that dual depletion of EED and RNF2 exhibits the same DNA hypomethylation phenotype as that of RNF2 single knockdown suggests that PRC2 promotes this balanced epigenetic signature by inhibiting PRC1's role in promoting DNA methylation (Fig. 7B,iii). Given that loci with dual H3K27me3 and H2AK119ub marks are most impacted by DNA methylation changes in our PRC1 or PRC2 function-depleted cells, we suspect that this canonical method of PRC1 recruitment via PRC2 may be an important mechanism for establishing the precise PRC2 – PRC1 control over DNA methylation deposition. Evidence that RNF2/H2AK119ub promotes methylation at many of the same promoters protected from hypermethylation by PRC2/H3K27me3 evokes a model for why select loci become hypermethylated, while the remainder of the genome undergoes hypomethylation in tumors (Fig. 7).

The herein identified roles of PRC2 and MLL/COMPASS in repressing DNA methylation deposition may represent key mechanisms in preventing the aberrant DNA hypermethylation events that typify human cancer given that PRC2- and MLL/COMPASS-protected loci are frequently hypermethylated in tumor cells. Future studies that determine whether this novel PRC2 regulatory function causes DNA hypermethylation in cancers with mutations that deplete genomic H3K27me3, such as H3.3 K27M mutant glioblastomas or myeloid leukemias and lymphomas with EZH2 loss of function mutations, are clearly warranted. For such tumors, aberrant DNA methylation may represent a major or early mechanism driving cancer development and these tumors might be particularly good candidates for emerging epigenetic therapies. For cancers lacking identifiable mutations in direct regulators of H3K27me3 (i.e., cancers with EZH2 gain or loss of function or H3.3 K27M mutations), H3K27me3-patterning mechanisms might be responsible for driving aberrant DNA hypermethylation and directly or indirectly tumor initiation or promotion. Genes or pathways that stabilize or maintain H3K27me3 during cell replication therefore represent novel tumor suppressor genes that might act to restrict cancer development by inhibiting aberrant DNA hypermethylation of PRC2-regulated loci.

## MATERIALS AND METHODS

### Cell culture, siRNA transfections, and extractions

NCCIT cells (from ATCC) were grown in McCoy's 5A medium as described [17]. On-TARGETplus SMARTpools (Dharmacon, Thermo Scientific) composed of a mixture of 4 individual siRNAs targeting a single gene were used against *WDR5* (L-013383-00), *EED* (L-017581-00), *RNF2* (L-006556-00), and *CBX4* (L-008356-00) in separate experiments and a combination of *EED* and *RNF2* pools together for the siEED+siRNF2 experiment. SiRNA transfection with a negative control non-targeting siRNA (D-001206-13-20; Dharmacon, Thermo Scientific) was performed in parallel. For siRNA transfections, 4.5 × 10^4^ NCCIT cells were seeded to each well of a 6-well plate. At 24 and 48 hours post seeding, cells were transfected using PepMute siRNA transfection reagent (SignaGen) prepared according to the manufacturer's protocol. Fresh growth medium (900 μl) was added to cells 30 minutes prior to addition of 100 μl of transfection reagent mix. The siRNA transfection mix for single targets contained 100 μl of PepMute transfection buffer, 1 μl of 40 μM siRNA pool, and 1.5 μl of PepMute reagent. For double EED and RNF2 siRNA targeting, a transfection mix with 100 μl of PepMute transfection buffer, 1 μl of 40 μM siRNA pool for each siRNA pool, and 3 μl of PepMute reagent was used. Fresh media was added to cells at 72 hours post-seeding, and cells were harvested at 96 hours post-seeding. Total RNA was extracted by Trizol homogenization and purified according to the manufacturer's protocol (Life Technologies). Genomic DNA was extracted by proteinase K digestion and phenol:chloroform extraction as described [38]. Histone acid extracts prepared in 0.1 N HCl and buffered with 100 mM Tris pH 9.0 were used for histone mark western blots and RNF2 western blot; whole cell extracts prepared in RIPA buffer with protease inhibitors were used for WDR5, KU70, CBX4, and PCNA western blots.

### Experimental validation by QRT-PCR expression analysis and western blotting

CDNA synthesis, QRT-PCR, and data analysis was performed as described previously [39]. QRT-PCR primers were designed and selected for optimal efficiency based on their performance with a standard curve of cDNA template. QRT-PCR was performed with at least three replicates. Primer sequences are listed in Supplementary Table 1. Antibodies used for western blotting were: H3K4me3 (Active Motif 39159), H3K4me2 (Millipore 07-030), H3K4me1 (Abcam ab8895), Histone H3 (Abcam ab1791), WDR5 (Bethyl A302-430A), KU70 (Santa Cruz sc9033), H3K27me3 (Millipore 07-449), H3K27me2 (Abcam ab24684), H3K27me1 (Millipore 07-448), H2AK119ub (Millipore 05-678), RNF2 (a gift from H. Koseki), CBX4 (Santa Cruz sc19299), and PCNA (Santa Cruz sc56).

### Affinity-based capture of 5mC and sequencing library preparation

Prior to affinity pull-downs, aliquots of 5 μg of genomic DNA in 130 μl TE were sheared to less than 400 bp on a Covaris S220 focused-ultrasonicator according to the manufacturer's instructions. Sheared samples were ethanol precipitated and resuspended in TE buffer. Two micrograms of sheared DNA was used as input for the MethylMagnet methylated-CpG DNA isolation kit according to the manufacturer's instructions (Ribomed) and reactions were performed in quadruplicate for each sample. DNA sequencing libraries were generated from the 5mC captured DNA and sequenced as described [17]. Libraries were sequenced on an Illumina HiSeq2000 (50 bp read length) at the Tufts University Genomics Core Facility.

### Data analysis

Raw sequencing reads were mapped to the UCSC human genome hg19 build using BWA V0.5.9 [40] with a default parameter setting. Multiply mapped reads and uniquely mapped reads with mismatches and indels > 5% of read lengths were filtered out. SICER V1.1 [41] was used to identify peaks in a sample and differentially enriched regions between two samples relative to an input with the following parameters: redundancy allowed = 1, window size = 200, fragment size = 300, effective genome size = 0.854, gap size = 600, E-value = 1000, false discovery rate = 0.01. In-house scripts annotated peaks and differentially enriched regions with RefSeq and CGIs in the UCSC genome browser [42], and classified them as promoter (−1kbp - +1 for TSS), body, and 3' end (TTS + 1kbp). In some cases, gene bodies were further classified into 5' UTR, exon, protein coding exon, 3' UTR, and intron. Genes were also stratified based on their promoter CpG density (high CpG density-HCP, intermediate CpG density-ICP, and low CpG density-LCP) using the criteria in [43]. In this classification, HCP are ‘strong’ CGIs while ICP are ‘weak’ CGIs. LCPs are a distinct class. Gene lists in promoters and bodies were analyzed using in-house scripts via the DAVID server (default settings) for functional annotation using gene ontologies and pathways [44]. After discarding more than two reads mapping to the same location, mapped reads were lengthened to the 3'-end to reflect their original length, and counted based on their midpoint for genomic features such as genes, CGIs, and repeats. A genomic feature was binned by relative positions including upstream and downstream regions. Different numbers of mapped reads per sample were taken into account by calculating FPKM (fragments per kilobase per million fragments mapped). To illustrate the change in tag density around genes, a relative length window for gene bodies was used and the average of normalized read coverage in a window was measured. Genes with differentially methylated promoters in each sample are listed in Supplementary Table 2. Gene expression microarray analysis was performed as described in [45]. Differentially expressed genes are listed in Supplementary Table 3.

### Infinium 450k and MeDIP-qPCR analysis

Genomic DNA was bisulfite treated and applied to the Infinium HumanMethylation450 BeadChip array [30] according to Illumina's protocols. Hybridization-based fluorescence intensity signals were read by the Illumina BeadStation GX scanner. Methylation profiling was performed with Illumina's GenomeStudio methylation module software. The ratio of fluorescent signals from the methylated alleles to the sum of the signals from all methylated and unmethylated alleles was derived to determine methylation beta values at all interrogated sites. Beta values from the siWDR5 and siNTC samples were compared, and differences of > 0.2 or < −0.2 and with p-values < 0.05 were the criteria used for differential methylation. Gene promoters (defined as the −1700 bp upstream of the TSS) containing one or more differentially methylated probes were classified as hypo- or hypermethylated.

MeDIP-qPCR. Five micrograms of RNA-free genomic DNA in 130 μl of TE buffer was sonicated to an average size of 400bp using a Covaris S220 sonicator. The DNA fragment sizes of 300 – 500bp were confirmed by agarose gel electrophoresis. DNA (2.5 μg) was added to MeDIP buffer (10 mM sodium phosphate (pH 7.0), 140 mM NaCl, 0.05% Triton x-100) and 10% of the volume was removed and kept as input in elution buffer (10 mM Tris-HCl (pH 8.0), 10 mM EDTA, 150mM NaCl, 5 mM DTT, 1% SDS) at -20°C. To the remainder of the sample, 1 μg of mouse monoclonal 5mC antibody (Diagenode, clone 33D3; catalog # C15200081-100) was added and incubated with rotation for three hours at 4°C. Three microliters of bridging antibody (Active Motif, catalog #53017) and 20 μl of pre-washed protein-G agarose beads were added, and rotated at 4°C overnight. The beads were washed three times with MeDIP buffer and two times with TE buffer, and the specifically bound DNA fragments were eluted twice with 50 μl of elution buffer at 65°C for 10 minutes. After purification with the MinElute PCR purification kit (Qiagen, catalog # 28004), the immunoprecipitated DNA and the input were used as templates for real-time qPCR for quantifying enrichment of specific loci defined in the 5mC-seq assays using the primers listed in Supplemental Table 1. The qPCR was performed with SYBR Green Supermix (Bio-Rad, catalog # 172-5274) using a C1000 Touch Thermal Cycler (with CFX96 real time system). PCR conditions were as follows: 95°C for 30 s; 95°C 5 s followed by 60°C 20 s for 40 cycles. Enrichment of methylation was calculated based on the formula: enrichment relative to input=2^-(sample aver Ct-Input aver Ct).

### Gene ontology analysis and statistical methods for data set comparisons

Ontology analysis was performed using the functional annotation tool within the DAVID bioinformatics database [44, 46]. Fisher's Exact test with a two-tailed p-value calculation was used for testing the significance of data set comparisons as described previously for similar data sets [47]. For added stringency, a modified EASE score was applied to all Fisher Exact tests [44, 46].

### Data access

Sequencing and expression microarray data has been deposited into the NCBI Gene Expression Omnibus database under accession GSE56539.

## SUPPLEMENTARY INFORMATION FIGURES AND TABLES








