# Supplementary material for: Impact of human MLL/COMPASS and polycomb complexes on the DNA methylome
Source: Oncotarget. 2014 Jul 14;5(15):6338–52. doi: 10.18632/oncotarget.2215 (PMC4171634; doi:10.18632/oncotarget.2215)
Supplement: Supplementary file 1 [file oncotarget-05-6338-s001.pdf]

Impact of human MLL/COMPASS and polycomb complexes on the DNA methylome

Supplementary Information

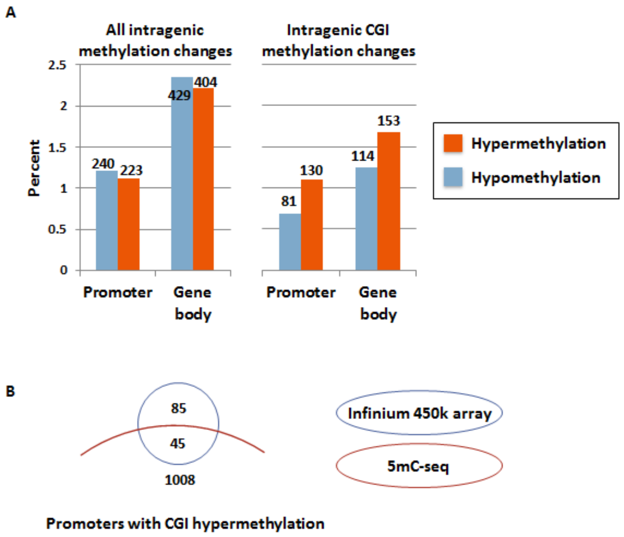

Supplementary Figure 1: Infinium 450k array-based confirmation of 5mC-seq DNA methylation results for siWDR5-treated cells. (A) Bar graphs showing the percentage of genes with differentially methylated probes in promoters and gene bodies as detected in siWDR5 cells, relative to the siNTC control, on the Infinium 450k array. All differentially methylated promoters and genes are shown on the left, and differentially methylated regions in CpG islands are shown on the right. Numbers represent the total number of genes. (B) Venn diagram illustrating the overlap between hypermethylated CGI promoters detected by 5mC-seq and the Infinium 450k array analyses after WDR5 depletion relative to the NTC control. Significance of overlap is $p < 0.0001$.

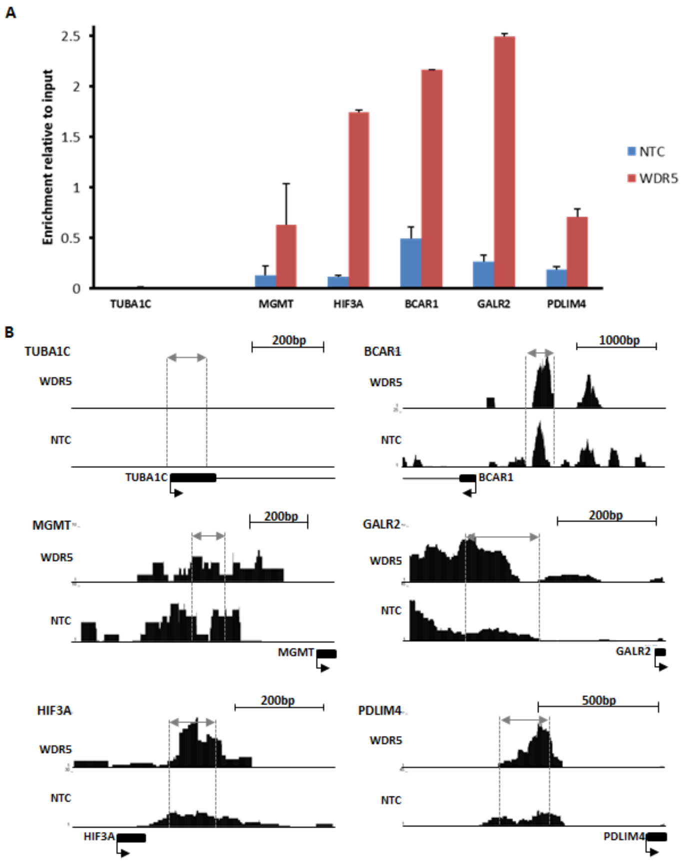

Supplementary Figure 2: Independent confirmation of 5mC-seq results by MeDIP-qPCR. (A) Detection of enrichment for each of the loci indicated by quantitative PCR following MeDIP. DNA from siNTC and siWDR5 knockdown NCCIT cells was used for MeDIP reactions. Each primer is run in triplicate with the error bar indicating the standard deviation (and set relative to input at 1.0, which is 10% of the IP'd sample). (B) 5mC-seq results as genome browser windows for each of the regions analyzed by qPCR in part A to demonstrate reproducibility between the two methods. The top track is the siWDR5 depletion and the bottom track is the siNTC control depletion. Gene features are shown below the browser tracks. Double-sided gray arrows indicate location of the MeDIP primers.

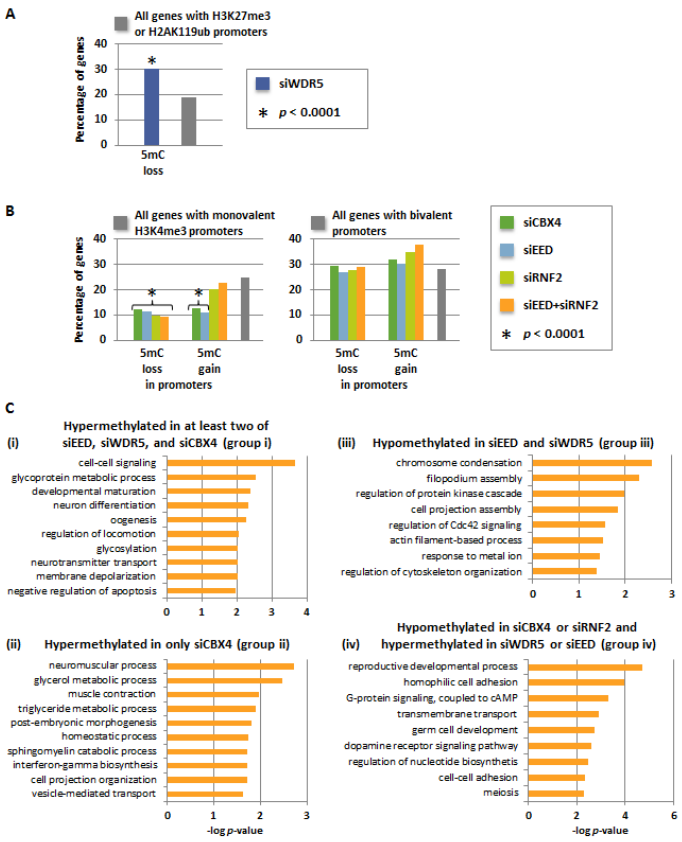

Supplementary Figure 3: Subsets of promoters targeted for DNA methylation control by PRC1 and PRC2. (A) Promoters hypomethylated under WDR5 depletion conditions (n=2054) were compared to those promoters containing PcG marks H3K27me3 and/or H2AK119ub, but lacking H3K4me3. The percentage of hypomethylated promoters overlapping PcG-marked promoters is shown (blue bar) in relation to the total percentage of PRC-marked promoters in the genome (grey bar). (B) Genes with promoter methylation changes after siRNA treatments were compared with the subsets of H3K4me3-monovalent promoters or H3K4me3+H3K27me3 bivalent promoters in NCCIT cells (not subject to siRNA depletion). Shown are the percentage of genes with differential methylation that are also H3K4me3-monovalent (left) or bivalent (right), and the percentage of all H3K4me3 monovalent and bivalent promoters in the genome (total number of genes = 23218). Promoters with methylation changes that are underrepresented among those with the given histone mark are designated with * when $p < 0.0001$. (C) Ontology analysis for gene groups of interest depicted in the hierarchical clustering of Figure 5B.

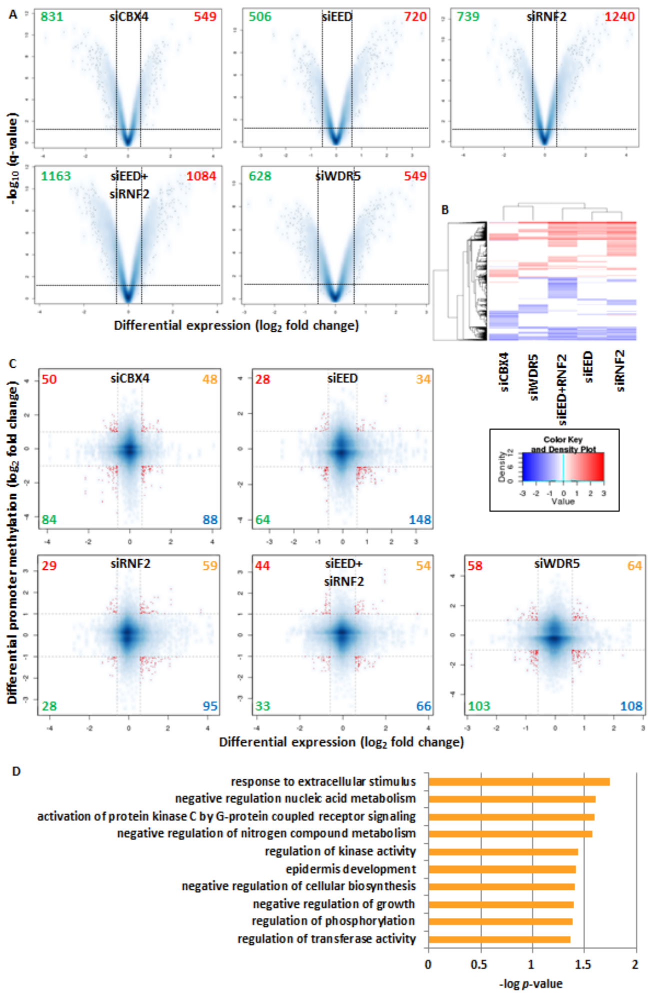

Supplementary Figure 4: Impact of MLL/COMPASS and PcG complexes on gene expression and links to DNA methylation. (A) Volcano plots representing expression changes on the x-axis, stratified by q-value on the y-axis. Vertical dotted lines represent limits for 1.5-fold increased and decreased expression. Horizontal dotted lines represents the limit for q-value = 0.5. Number of genes with decreased or increased expression with > 1.5-fold change and q-value < 0.05 are represented by the green and red numbers, respectively. (B) Heat map of hierarchical clustering of differentially expressed genes shown as $\log_2$ methylation changes. (C) Scatterplots illustrating differential expression (x-axis) versus differential promoter methylation (y-axis). Vertical and horizontal lines demarcate the limits for 1.5-fold expression changes and 2-fold promoter methylation changes, respectively. Numbers in each quadrant represent the number of genes that exceed both limits for expression and methylation changes. (D) Ontology analysis of the subset of genes that become hypomethylated and transcriptionally upregulated upon depletion of EED or RNF2 function.
